# On a Death from Heart Disease, Occurring in a Swimming Bath

**Published:** 1851-02

**Authors:** Wm. James Jones

**Affiliations:** London


*On a Death from Heart-disease occurring in a swimming bath.* By Wm. James Jones, Esq., M.R.C.S., London.—Having for many years taken great interest in the art of swimming, and having also, as chairman of a long-established swimming club, had great opportunities of observing the effects produced upon the system by the healthful habit of tepid bathing, I am induced to forward you the results of an autopsy on the body of a gentleman, who, without apparent previous illness, died suddenly whilst bathing in the Holborn baths. I consider that by so doing I shall remove, in some measure, the prejudice connected with this most useful and necessary art, and at the same time offer an explanation of the very many sudden deaths occurring to able swimmers in the water, wherein the loss of life has been erroneously attribnted to cramp, exhaustion, &c., when, in reality, sudden death would have occurred, and does occur, under other circumstances, where any great muscular exertion is used by patients laboring under organic disease of the heart, lungs, or brain, more particularly of the heart.

On the 28th of last August I was hastily summoned to the Holborn baths, the messenger informing me that a gentleman was drowned there. On reaching the patient, I found a young man, about twenty years of age, in a warm bath, and whilst endeavoring to glean some particulars of the accident, I made use of the usual method adopted for the resuscitation of the drowned. These means were put in force for half an hour, during which period I felt convinced, from the testimony of the bystanders, that the sufferer could not have been submerged more than half a minute. That such an accident should occur to an able swimmer I thought improbable, and conceiving that death was produced from asphyxia, from cerebral congestion, through partial intoxication, or a full stomach, I opened the jugular vein, from which flowed a small quantity of black blood. All subsequent attempts to restore by artificial respiration, friction, &c., proving fruitless, the deceased was, on the following day, examined by me, under an order from Mr. H. M. Wakley, the deputy-coroner for Middlesex.

I may observe that the deceased plunged into the water from a height of four feet, that he dived about twelve yards, that he subsequently swam twenty, and then was seen to sink like a stone to the bottom of the bath.

*Autopsy.*—The body externally presented nothing peculiar; was tolerably fat, well-formed, with the neck somewhat shorter than usual. There was a band of tape round the left leg, which was said to have been applied by the deceased in consequence of a varicose vein having bled sometime previously. There was cataract of the right eye, which I subsequently learned was congenital. The pupil of the left eye not much dilated, and the face of a purplish color. On opening the chest and abdomen, I was struck by the unusually distended state of the stomach and flatus, which pressed with great force against the diaphragm, and must materially have prevented its descent in inspiration. It at the same time contained a large quantity of half-digested food, and *no water;* it was healthy. The lungs were healthy, and not at all congested; that of the left side was attached to the costal pleura by very many strong adhesions; they contained no water, and the trachea, bronchi, and air-cells held but very little frothy mucus. The pericardium was firmly attached to the heart by a succession of adhesions as to present what the older pathologists considered a congenital absence of this bag, the pericardial cavity being completely obliterated, and the membrane itself so adherent as to be removed with difficulty without a scalpel. The heart was tightly bound down by its covering, both its cavities being full of black unoxygenated blood in a liquid state; the right auricle was greatly dilated, so as to represent a perfect aneurism of that organ. The brain was healthy, and did not present any congested condition, except in the meningeal veins; there was some fluid in the lateral ventricles, but not much.

With these appearances there could be no question as to the cause of death, in the absence of the usual signs of suffocation; and the case is remarkable, inasmuch as it shows the possibility of the existence of very extensive lesions, both of the heart and its coverings, without the suspicion of any disease at all existing in the minds either of patient or friends during life. Such an attachment of the pericardium to the heart could be but the result of frequent attacks of pericarditis, and it would appear, from the testimony of deceased's father, that his son had on one occasion only complained of slight pain in the chest, which did not prevent him from following his usual avocation, and that, in fact, he never had a day's illness in his life. I may observe that his father had been educated for the medical profession, and was a very intelligent, well-informed man.

A gentleman who saw him before he bathed, and who was both a barrister and surgeon, noticed the peculiar color of his face, which he compared to that of blotting-paper.

The beautiful attempt of Nature to make up for the restricted condition of the heart, by a dilatation of the right auricle, may, perhaps, explain the slight inconvenience felt from so extraordinary a state of its bag; and in a medico-legal point of view, various interesting questions might be suggested under such peculiar circumstances.—*Ibid.*

